# Role of EZH2-mediated epigenetic modification on vascular smooth muscle in cardiovascular diseases: A mini-review

**DOI:** 10.3389/fphar.2024.1416992

**Published:** 2024-06-27

**Authors:** Haiyan Luo, Yao Li, Honghu Song, Kui Zhao, Wenlin Li, Hailan Hong, Yun-Ting Wang, Luming Qi, Yang Zhang

**Affiliations:** ^1^ School of Health Preservation and Rehabilitation, Chengdu University of Traditional Chinese Medicine, Chengdu, Sichuan, China; ^2^ Institute of Traditional Chinese Medicine Health Industry, China Academy of Chinese Medical Sciences, Nanchang, China; ^3^ Jiangxi Province Key Laboratory of Traditional Chinese Medicine Pharmacology, Nanchang, China; ^4^ College of Material Science and Chemical Engineering, Southwest Forestry University, Kunming, Yunnan, China; ^5^ Center for Quality Evaluation and Research in Higher Education, Nanjing University of Chinese Medicine, Nanjing, China; ^6^ Department of Pharmacological and Pharmaceutical Sciences, College of Pharmacy, University of Houston, Houston, TX, United States

**Keywords:** epigenetic modification, EZH2, vascular smooth muscle cells, cardiovascular disease, atherosclerosis, hypertension

## Abstract

Vascular smooth muscle cells (VSMCs) are integral to the pathophysiology of cardiovascular diseases (CVDs). Enhancer of zeste homolog 2 (EZH2), a histone methyltransferase, plays a crucial role in epigenetic regulation of VSMCs gene expression. Emerging researches suggest that EZH2 has a dual role in VSMCs, contingent on the pathological context of specific CVDs. This mini-review synthesizes the current knowledge on the mechanisms by which EZH2 regulates VSMC proliferation, migration and survival in the context of CVDs. The goal is to underscore the potential of EZH2 as a therapeutic target for CVDs treatment. Modulating EZH2 and its associated epigenetic pathways in VSMCs could potentially ameliorate vascular remodeling, a key factor in the progression of many CVDs. Despite the promising outlook, further investigation is warranted to elucidate the epigenetic mechanisms mediated by EZH2 in VSMCs, which may pave the way for novel epigenetic therapies for conditions such as atherosclerosis and hypertension.

## 1 Introduction

Cardiovascular diseases (CVDs) have long posed a significant global health challenge, characterized by for high incidence, disability, and mortality rates. According to the World Health Statistics Report published by the World Health Organization in 2017, CVDs accounted for a staggering 17.7 million deaths globally in 2015, representing 44% of all deaths worldwide. This figure includes more than half of total deaths in European region (Machado et al., 2020). Despite advancements in existing treatments that have improved patient quality of life, the morbidity and mortality associated with CVDs continue to rise in correlation with societal progress and changes in human lifestyles. Thus, the pursuit of new therapeutic targets is both urgent and critical.

Vascular smooth muscle cells (VSMCs) are predominantly located within the vessel wall and are essential for regulating vascular contraction, thus maintaining vascular homeostasis. In response to vascular injury or stimulation by bioactive substances such as nitric oxide products, angiotensin II (Ang II), and platelet-derived growth factor, VSMCs proliferate and migrate. This process is a key mechanism underlying blood vessel wall thickening, lumen narrowing, and vascular remodeling (Ren et al., 2017; Zhang et al., 2018; Wang et al., 2019; Wang et al., 2019; Yuan et al., 2020). VSMCs can also undergo phenotypic changes in various environmental contexts, which significantly impact the development of atherosclerosis (AS) (Basatemur et al., 2019). During vascular injury and inflammation, VSMCs proliferate and migrate to form a fibrous cap, eventually invading the core of the plaque, thereby advancing AS (Misra et al., 2018). Consequently, the phenotype of VSMCs, influenced by the immune milieu, plays a crucial role in AS etiology and subsequent CVDs (Ramel et al., 2019). The apoptosis of VSMCs, contributing to fibrous cap and plaque instability, activation of calcification (Proudfoot et al., 2000) and immune system (Schrijvers et al., 2005), further exacerbate AS development. Additionally, abnormal VSMC proliferation, migration, and apoptosis are pivotal in other CVDs, including hypertension, aortic dissection (AD), aortic aneurysm (AA), and coronary heart disease (CHD) (Wang and Chen, 2020). These insights underscore the critical role of VSMCs in the pathophysiology of CVDs and provide new perspectives for understanding and treating these diseases.

Enhancer of zeste homolog 2 (EZH2) is a methyltransferase from the Polycomb gene family, significantly impacting VSMC proliferation and differentiation. EZH2 regulates these processes by modifying histone methylation on microRNAs and the N-terminal tail of the 27th amino acid, lysine, on core histone H3 (H3K27me3) (Zhang et al., 2021; Li et al., 2022; Zhong et al., 2022). Furthermore, EZH2 influences VSMC contraction and relaxation by modulating the expression of critical genes such as those encoding calcium channels and actin. Inhibitors of EZH2 have shown considerable potential in enhancing vascular function and treating CVDs, including hypertension and AS. Therefore, a comprehensive understanding of the mechanisms underlying EZH2-targeted regulation of VSMC function in CVDs is imperative for developing novel therapeutic strategies. This mini-review aims to summarize the research progress on EZH2-mediated epigenetic modifications in VSMCs in the context of CVDs, providing a reference for the development of EZH2-targeted therapies.

## 2 CVDs from the EZH2 perspective

### 2.1 Structure and function of EZH2

**FIGURE 1:**
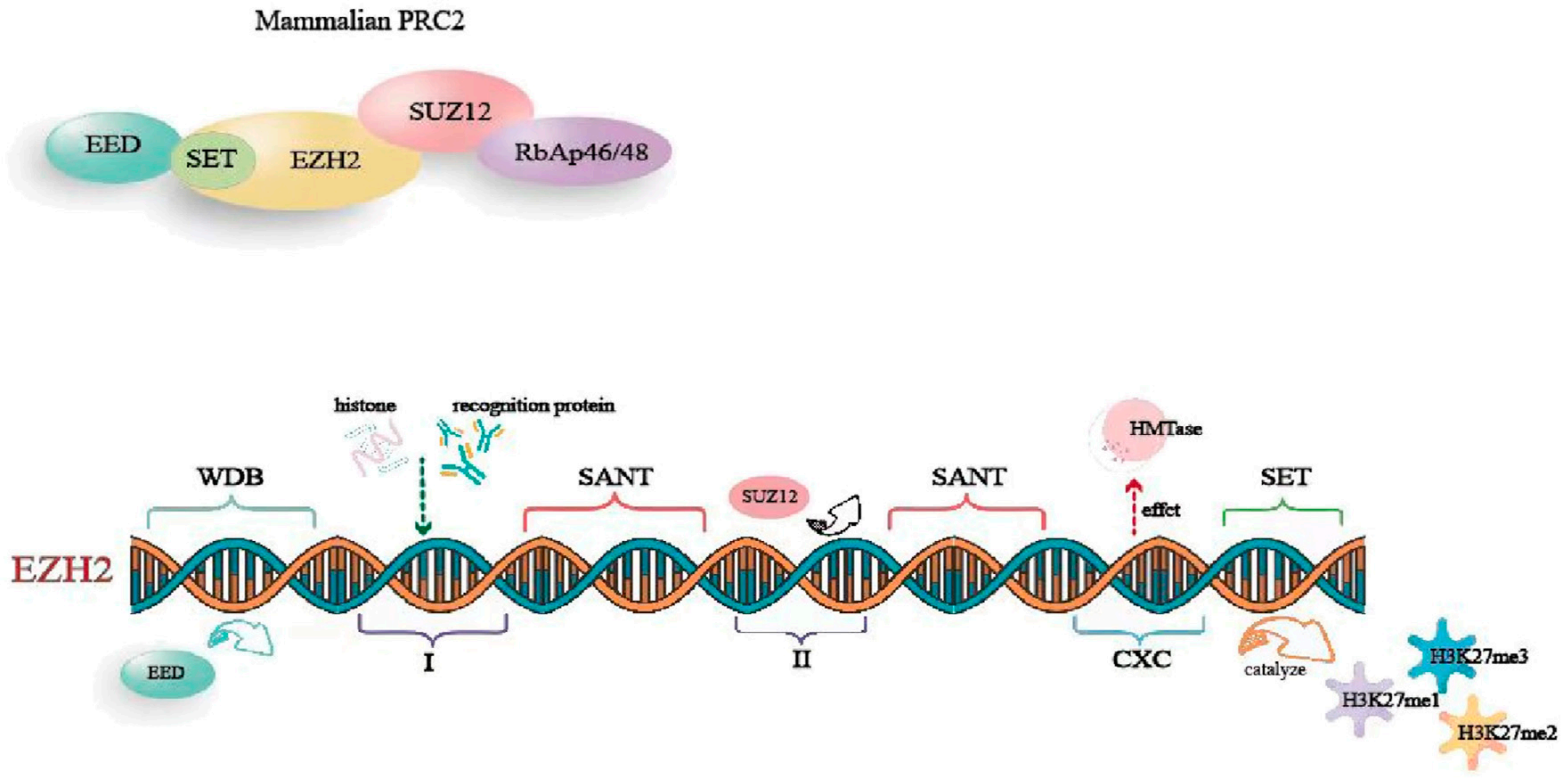
The basic structure of EZH2.

EZH2 is a key component of the polycomb repressive complex 2 (PRC2) (Heng et al., 2023). It interacts with several proteins, including suppressor of zeste 12 (SUZ12), embryonic ectoderm development protein (EED), retinoblastoma-binding protein 4/7 (RBBP4/7), and adipocyte enhancer-binding protein 2 (AEBP2), forming PRC2 through domains WD-40, I, and II. This complex plays a critical role in initiating transcriptional repression. The human EZH2 gene is located in the q35 region of chromosome 7 and consists of 20 exons encoding 747 amino acids (Pingping et al., 2022). EZH2 comprises five primary domains: 1) WD-40 binding domain, which mediates EED binding; 2) Domains I and II, in which domain I binds to PHF1 and domain Ⅱ binds to SUZ12; 3) two SANT domains, which involve in recruiting other chromatin remodeling factors for histone binding; 4) The cysteine-rich domain (CXC), which influences HMTase activity and participates in EZH2 stability and subcellular localization; 5) The SET domain, which is a conserved catalytic domain at the C-terminal, catalyzing the production of mono-, di-, and trimethylated H3K27 (H3K27me1, H3K27me2, and H3K27me3) (Pingping et al., 2022; Heng et al., 2023) (Figure 1).

Initially, studies focused on EZH2-mediated gene silencing through its histone methyltransferase activity in a PRC2-dependent manner (Heng et al., 2023). By trimethylating the lysine 27 site of histone H3 to form the H3K27me3, EZH2 regulates various physiological and pathological processes, including cell proliferation, differentiation, aging and tumorigenesis. Recent research has unveiled that EZH2 is a multifunctional molecule. At transcriptional level, EZH2 can modify non-histone proteins via methylation, thereby altering their functions. Additionally, EZH2 can act as a protein scaffold, recruiting different transcription factors to form large complexes that influence their transcriptional activity (Pingping et al., 2022). Beyond transcriptional regulation, EZH2 also regulates cell migration, activates signaling pathways (Adamik et al., 2020), and interacts with RNA (Kaneko et al., 2010), independently of transcriptional functions. Today, EZH2 is recognized for its significant regulatory roles in various biological events, including cardiovascular development, tumorigenesis and metastasis, and degenerative aging (Mahara et al., 2016; Yuan et al., 2021; Chao et al., 2022; Pingping et al., 2022).

### 2.2 Development of atherosclerosis from the EZH2 perspective

Atherosclerosis (AS) is a typical CVDs characterized by chronic inflammatory vascular lesion (Herrington et al., 2016). The proliferation, migration, apoptosis, and phenotypic transformation of VSMCs are the critical processes driving AS development (Bennett et al., 2016; Wu et al., 2017; Wang et al., 2019). The histone methyltransferase EZH2 in VSMCs plays a significant role in the pathogenesis of AS by affecting epigenetic regulation. Investigating EZH2 in VSMCs offers new perspectives on the pathophysiology and treatment of AS.

During AS formation, vascular injury is a crucial factor in initiating lesion development. Studies have shown that vascular injury increases the expression of EZH2 and H3K27me3 in smooth muscle cells of carotid arteries. Growth medium and platelet-derived growth factor BB also enhance the expression of EZH2 and H3K27me3 in cultured VSMCs (Liang et al., 2019). This process inhibits the expression of VSMCs-specific marker smooth muscle protein 22-α (SM-22α) (Liang et al., 2019) affecting the maintenance of VSMCs phenotype and resulting in proliferative and migratory phenotypes. This phenotype switch promotes neointima formation and exacerbates AS development (Liu et al., 2016). SM-22α knockout mice, which lack this key phenotype maintenance factor, are more prone to phenotypic switching, leading to an increased incidence of AS (Shen et al., 2010). These findings suggest that EZH2 upregulation is involved in injury-triggered VSMCs phenotypic transformation promoting AS development by affecting the expression of H3K27me3 and SM-22α.

Inflammatory responses are another key factor in AS formation. EZH2-regulated signaling pathways play an important role in this process. Specifically, expression of EZH2 is upregulated in inflammatory environments, leading to increased H3K27me3 levels, which suppress the expression of SM-22α. Conversely, the anti-inflammatory effect of the histone deacetylase Silent Information Regulator 1 (SIRT1) is dependent on the presence of SM-22α. Thus, the upregulation of EZH2 inhibits the anti-inflammatory function of SIRT1. In turn, SIRT1 can remove the acetyl modification of SM-22α by EZH2 through deacetylation, thereby increasing SM-22α expression and exerting anti-inflammatory effects (Shu et al., 2017). This EZH2-SIRT1-SM-22α regulatory loop is crucial in the onset and progression of AS. Overall, EZH2 in VSMCs promotes AS development by influencing cell proliferation, migration, apoptosis, and phenotypic transformation during vascular injury and inflammation.

The microRNA miR-139-5p is a highly conserved small non-coding RNA that mediates the response of mRNA transcripts to various signaling pathways in cells. Its direct target gene, signal transducer and activator of transcription (STAT1), can reduce angiogenesis and thus inhibit AS development (Zheng et al., 2021). In VSMCs, EZH2 can bind to H3K27me3, the promoter of miR-139-5p, down-regulating miR-139-5p expression and increasing STAT1 signaling expression, as verified in arterial tissues of AS patients (Zheng et al., 2021). Thus, EZH2 can influence AS development by regulating miR-139-5p/STAT1 signaling pathway.

Moreover, proprotein convertase subtilisin/kexin type 9 (PCSK9), a serine protease produced by the liver, has been shown to be involved in the development of dyslipidemia and CVDs caused by AS (Ragusa et al., 2021; Katsuki et al., 2022). PCSK9 inhibitors are used for the treatment of AS or other CVDs. Small nucleolar RNA Host Gene 16 (SNHG16), a downstream effector of PCSK9, promotes VSMC proliferation, migration, and foam cell formation, and this effect can be reversed by TNF receptor associated factor 5 (TRAF5). SNHG16 inhibits the reversal pathway of TRAF5 by recruiting EZH2, which promotes AS development (Liu et al., 2023). Therefore, EZH2 plays a crucial role in SNHG16-mediated TRAF5-regulated VSMC proliferation, migration and foam cell formation pathway, further influencing AS progression.

In advanced AS and vascular inflammation, the pro-fibrotic molecule cellular communication network factor 2 (CCN2) is highly expressed, and its expression is further upregulated by EZH2 under hypoxic conditions. In contrast, the polymerase delta-interacting protein 2 (Poldip2) inhibits CCN2 expression, but itself is inhibited by EZH2 under hypoxic conditions (Paredes et al., 2023). Thus, EZH2 can inhibit Poldip2 expression, increase CCN2 expression, and exacerbate fibrosis caused by AS under hypoxic conditions. Inhibition of EZH2 can mitigate AS development by ensuring the normal expression of Poldip2 and its downstream CCN2.

**FIGURE 2:**
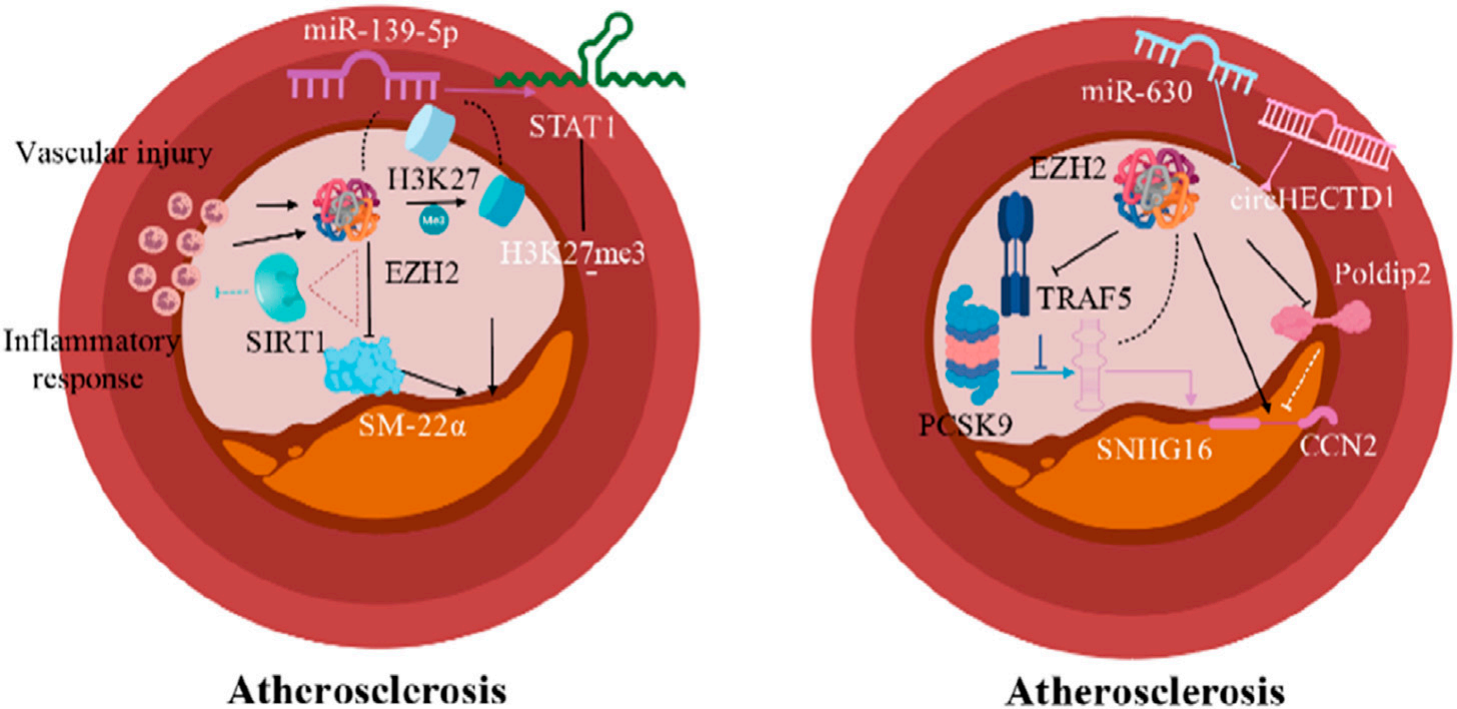
Epigenetic modification mechanisms of EZH2 in AS (Arrows indicate facilitation of this molecular mechanism, and horizontal lines indicate inhibition of this molecular mechanism).

Additionally, the circHECTD1 gene promotes VSMC proliferation and migration (Feng et al., 2023). Silencing EZH2 in VSMCs can reverse the proliferation enhancement effect of circHECTD1 on VSMCs. As an important histone modification regulator, EZH2 can be specifically targeted to inhibit circHECTD1 and reduce atherosclerotic plaque formation (Feng et al., 2023). Conversely, mechanical stretch (Zhong et al., 2022) and miR-630 overexpression (Miao et al., 2022) can inhibit EZH2 expression in VSMCs, reducing atherosclerotic lesions (Figure 2). Overall, EZH2 is involved in the pathogenesis of AS through multiple upstream and downstream pathways. Focusing on EZH2 targets in VSMCs holds promising prospects for exploring new directions for AS treatment.

### 2.3 Development of hypertension from the EZH2 perspective

Hypertension is a chronic diseases characterized by endothelial dysfunction, increased vasoconstriction, abnormal proliferation and migration of VSMCs and vascular remodeling (Intengan and Schiffrin, 2000; Savoia et al., 2011; Montezano et al., 2015). Vascular remodeling is persistent throughout the course of hypertension (Hayashi and Naiki, 2009) and serve as an important indicator of the hypertensive condition (Bakker et al., 2005). Dysfunction of VSMCs plays a key role in vascular remodeling. EZH2 influences the development of hypertension by regulating VSMC proliferation, migration and phenotypic transition. Therefore, an in-depth study of the mechanism of EZH2 in VSMCs dysfunction is necessary for better understand and treat hypertension.

MicroRNAs are known to regulate VSMC proliferation, migration and differentiation, processes highly correlated with vascular remodeling (Chen et al., 2018; Chen et al., 2018; Wang and Atanasov, 2019; Wang et al., 2019). For example, miR-26a can regulate EZH2 expression and play a protective role in vascular health (Zhang et al., 2021). Targeting EZH2 via modulating miR-26a expression can reduce VSMC proliferation and inhibit vascular remodeling in hypertension.

The vascular remodeling caused by abnormal VSMC proliferation and migration is also a critical event in the process in the onset and progression of pulmonary arterial hypertension (PAH) (Archer et al., 2010; Gladwin and Ghofrani, 2010), leading to vascular obstruction and destruction that adversely affect lung and heart function. Experimental studies have shown that EZH2 overexpression enhances proliferation, migration and apoptosis resistance of pulmonary artery VSMCs in the mice with hypoxia-mediated PAH (Aljubran et al., 2012; Saco et al., 2014). This suggests that EZH2 is an effective regulator of pulmonary artery VSMC homeostasis, with disruption in its expression influencing PAH development. Moreover, EZH2 and its downstream effectors could serve as therapeutic targets for combating pulmonary vascular remodeling (Habbout et al., 2021). Superoxide dismutase 2 (SOD2) deficiency, linked to PAH through redox homeostasis disruption and VSMC phenotypic transformation, is also influenced by EZH2 (Archer et al., 2010; Wang et al., 2021). Upregulation of EZH2 exacerbates PAH by promoting the transformation of pulmonary VSMC into a proliferative phenotype, thereby worsening vascular remodeling and hemodynamic changes in SOD2 deficiency mice (Wang et al., 2021). This implies a connection between EZH2’s role in VSMC phenotype transition and redox signaling (Wang et al., 2021).

Bone morphogenetic protein receptor type 2 (BMPR2) belongs to a family of genes involved in cell growth and differentiation (Soon et al., 2015; Orriols et al., 2017). Loss of BMPR2 function exacerbates PAH (Liu et al., 2017) with most PAH patients exhibiting significantly reduced BMPR2 expression (Atkinson et al., 2002; Morrell, 2010; Rabinovitch, 2012). Soon et al. demonstrated that BMPR2 deficiency promotes PAH development by decreasing superoxide dismutase 3 (SOD3) expression and enhancing inflammatory responses. Recent findings indicate that EZH2 interacts with switch-independent 3a (SIN3a) to regulate BMPR2 in human pulmonary artery VSMCs, impacting PAH development. SIN3a deficiency promotes proliferation and migration of pulmonary artery VSMCs, while its overexpression counteracts these effects. Specifically, overexpression of SIN3a reduces methylation of the BMPR2 promoter region in human pulmonary artery VSMCs, activating BMPR2 transcription (Soon et al., 2015). This suggests that SIN3a overexpression can inhibit EZH2-mediated proliferation of human pulmonary artery VSMCs. Since EZH2 suppresses target gene expression via H3K27me3 catalysis, SIN3a antagonizes EZH2’s function. Thus, the epigenetic regulation between SIN3a and EZH2 represent a critical node in controlling pulmonary artery VSMCs’s phenotype switch and growth. SIN3a modulates BMPR2 DNA methylation and expression by regulating EZH2 levels and decreasing H3K27me3 content. While EZH2 inhibits BMPR2 expression through H3K27me3, SIN3a opposes this function, upregulates BMPR2, and mitigates PAH (Bisserier et al., 2021). Therefore, targeting the combination of SIN3a and EZH2 could represent a novel therapeutic strategy for PAH.

### 2.4 Development of aortic dissection from the EZH2 perspective

Aortic dissection (AD) is a life-threatening cardiovascular emergency caused by a tear in the aorta’s lining or bleeding within the aortic wall, leading to the separation of its layers (Nienaber et al., 2016; Gawinecka et al., 2017). The key pathological features in AD patients include media degeneration, characterized by elastic fibers breakage and loss, VSMCs loss, and accumulation of mucopolysaccharides (Larson and Edwards, 1984; Zhu et al., 2006; Jiang et al., 2016). VSMC cycle arrest, apoptosis, necrosis and autophagic cell death are potential causes of VSMC loss in the aortic wall (Huang et al., 2015; Jia et al., 2015; Wang et al., 2016). Epigenetic regulatory factors present in VSMCs can effectively manage these processes.

Studies have shown that alkylation repair homologous protein 5 (ALKBH5), a key m6A demethylase, is highly expressed in the aortic tissue of AD patients, suggesting m6A modification’s involvement in AD progression (Wang et al., 2021). ALKBH5 exacerbates Ang-II-induced inflammatory responses and apoptosis in human aortic VSMCs. Long non-coding RNA (lncRNA)-TMPO-AS1, a downstream target of ALKBH5, affects AD progression partly by binding with EZH2 to epigenetically regulate interleukin-1 receptor-associated kinase 4 (IRAK4). EZH2 downregulates IRAK4, reducing ALKBH5 expression and alleviating AD disease (Wang et al., 2021). DNA methylome analysis reveals hypomethylation of EZH2 targets and overexpression of retinoic acid receptor alpha gene in AD patients (Pan et al., 2017). Additionally, cytosolic DNA through stimulator of interferon response CGAMP interactor 1 (STING-1) and interferon regulatory factor 3 (IRF3) signaling recruits EZH2, inducing H3K27me3 modification and driving VSMCs from a contractile to an inflammatory phenotype during AD formation (Chakraborty et al., 2023). Therefore, targeting EZH2 in the regulatory network for AD progression is promising for AD intervention.

Moderate autophagy is crucial for VSMC proliferation, migration, apoptosis and vascular remodeling (Grootaert et al., 2018), but excessive autophagy leads to VSMC loss. Inhibition or knockdown of EZH2 induces cell cycle arrest and autophagic cell death via MEK-ERK1/2 signaling pathway, contributing to VSMC loss and AD development, while EZH2 overexpression promotes proliferation and reduces autophagic cell death. Thus, EZH2 affects AD development by regulating VSMC autophagy (Li et al., 2018). Therefore, enhancing EZH2 function could be explored to reduce AD occurrence.

### 2.5 Development of AAA and TAA from the EZH2 perspective

EZH2 is also closely related to the occurrence and development of abdominal aortic aneurysm (AAA) and thoracic aortic aneurysm (TAA). Both AAA and TAA involve structural and functional disorders of the aorta, increasing the risk of aortic wall dilation and rupture. VSMCs dysfunction and pathological processes such as inflammation, immune response and extracellular matrix remodeling contribute to AAA formation (Wortmann et al., 2019). lncRNAs regulate VSMC proliferation, migration, and apoptosis, affecting AAA development (Wu et al., 2020). For example, lncRNAs like GAS5, H19, LCC-HLTF-5, and HIF1-α-AS1 are implicated in AAA development (Kumar et al., 2019). Retinal acid-induced gene I (RIG-I) gain-of-function variation leads to aortic and coronary artery calcification (Jang et al., 2015). EZH2 inhibits VSMC apoptosis by suppressing RIG-I signaling pathway independent of methylation, while GAS5 may promote VSMC apoptosis by inhibiting EZH2 expression (Le et al., 2021). Therefore, GAS5 may regulate AAA development through the EZH2/RIG-I axis, promoting apoptosis and accelerating the AAA development by inhibiting EZH2 expression.

**FIGURE 3:**
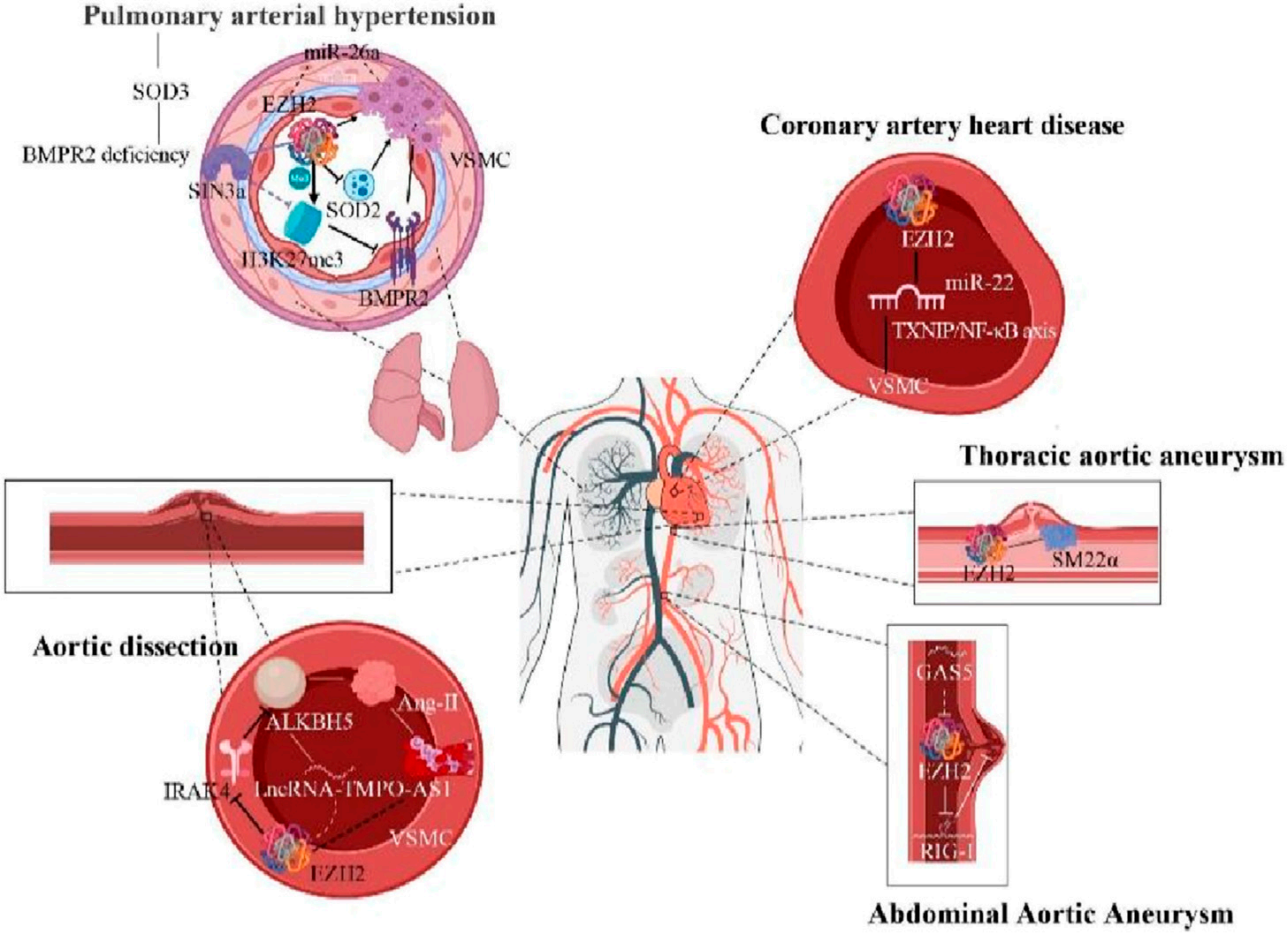
Epigenetic modification mechanisms of EZH2 in hypertension, AD, CHD, AAA, and TAA (Arrows indicate facilitation of this molecular mechanism, and horizontal lines indicate inhibition of this molecular mechanism).

Contractile proteins are crucial for VSMC cytoskeleton maintenance. Defects in contractile protein expression are identified TAA development (Cardenas et al., 2018). SM-22α, specifically expressed in contractile VSMCs, is essential for maintaining the VSMC contractile phenotype. SM-22α expression negatively correlates with aneurysm size, and EZH2 inhibition can enhance SM-22α expression. Targeting EZH2 with the small molecule inhibitor like GSK343 improves aortic function in Fbn1C1039G/+ mice by restoring contractile protein expression (Cardenas et al., 2018). Thus, VSMC contractile proteins play a key role in the maintaining aortic homeostasis and are potential targets for epigenetic modification in thoracic aortic disease (Cardenas et al., 2018). EZH2 maintains VSMC lineage and properties in TAA (Wang et al., 2010). Inhibiting EZH2 more effectively expresses VSMCs contractile proteins (Figure 3), presenting new therapeutic pathways for TAA and insights into clinical epigenetic events. Overall, EZH2 plays a significant role in AAA and TAA development, offering potential research and clinical applications as a therapeutic target.

### 2.6 Development of other CVDs from the EZH2 perspective

Coronary heart disease (CHD), or ischemic heart disease, results from myocardial ischemia and hypoxia due to coronary atherosclerosis. VSMC dysfunction significantly contributes to CHD pathogenesis. Downregulation of miR-22 in peripheral blood mononuclear cells corelates with CHD in patients (Chen et al., 2016). Overexpression of miR-22 reduces VSMC proliferation and migration, thereby inhibiting CHD development (Liu et al., 2020). EZH2 inhibits miR-22 transcription, leading to an imbalance in the TXNIP/NF-κB axis, a classical pathway contributes to CHD development (Liu et al., 2020). Thus, EZH2 can affect TXNIP/NF-κB signaling pathway by regulating miR-22 expression, offering a new potential therapeutic target for CHD treatment.

## 3 Discussion

EZH2 has garnered significant attention as a therapeutic target in epigenetics for various diseases. However, its regulatory role in VSMCs in CVDs is often overlooked. As an epigenetic regulator, EZH2 in VSMCs is crucial for maintaining the balance of several signaling pathways in CVDs.

This review discusses the recent advances in understanding the role of EZH2 in preventing and treating CVDs by modulating the proliferation, migration and apoptosis of VSMCs. From an epigenetic perspective, EZH2 emerges as a potential target for CVDs therapy. Our analysis indicates that EZH2 exerts a bidirectional regulatory effect on the pathogenesis and progression of CVDs by affecting the signaling pathways within VSMCs.

Despite the varied drivers of VSMCs dysfunction, abnormal proliferation and migration processes are strongly linked to EZH2 expression. We propose that EZH2 inhibitor could serve as therapeutic strategies for conditions like restenosis after angioplasty, atherosclerosis, and vein graft intimal thickening by inhibiting post-surgical VSMC proliferation and migration, thereby reducing restenosis incidence. Preclinical studies have shown that the EZH2 inhibitor UNC1999 significantly suppresses PDGF-BB-induced VSMC proliferation and neointimal hyperplasia (Liang et al., 2019). VSMCs’ ability to dedifferentiate and switch phenotypes in response to environmental stimuli is critical hypertension pathophysiology (Liu et al., 2015). This phenotypic switch can be regulated by epigenetic modifications (Liu et al., 2015; Levy et al., 2017). Increased EZH2 expression promotes hyperproliferation and an anti-apoptotic phenotype in PAH-PASMC through both classical and non-classical mechanisms (Habbout et al., 2021). Another preclinical study demonstrated that the EZH2 inhibitor EPZ005687 ameliorates PAH and improves cardiovascular function in mice (Shi et al., 2018). Hence, targeting EZH2 in VSMCs maybe a promising approach for treating diseases like AS and hypertension characterized by abnormal VSMC proliferation, migration and vascular remodeling.

Conversely, EZH2 is also vital for VSMCs survival. The pathophysiological of AD is complex with VSMC dysfunction playing a crucial role (Liu et al., 2022). Studies have shown that EZH2 reduces autophagic cell death in aortic VSMCs by inhibiting autophagosomes formation through ATG5 and ATG7 repression, thereby supporting VSMC survival during AD (Li et al., 2018). Epigenetic modification-mediated VSMC dysfunction and extracellular matrix degradation are common in TAA and AAA (Michel et al., 2018). EZH2 overexpression promotes PASMC proliferation and migration while reducing apoptosis (Aljubran et al., 2012). EZH2 knockdown leads to VSMC loss (Li et al., 2018), which can accelerate AAA development (Siasos et al., 2015). Moreover, EZH2 overexpression can mitigate AAA progression by increasing H3K27me3 levels at annexin A6 (ANXA6) promoter, reducing ROS levels and VSMC senescence induced by Ang II (Li et al., 2022). Multiple gene products, particularly the VSMC contractile protein SM-22α, are deficient human and mouse TAA samples, and its loss is a directly linked to aneurysm progression and AD *in vivo*. EZH2 inhibition can improve SM-22α expression, potentially benefiting TAA conditions (Cardenas et al., 2018). Additionally, the activation of the STING-IRF3-EZH2 axis drives VSMCs from a contractile to an inflammatory phenotype during AD formation (Chakraborty et al., 2023). Thus, enhancing EZH2 function in VSMCs could be explored as a treatment for vascular diseases characterized by VSMC loss, such as AD, TAA and AAA.

Reprogramming somatic cells into induced cardiomyocytes (iCMs) is a promising regenerative medicine strategy for CVDs. Studies have shown that EZH2 acts as an epigenetic barrier during human iCMs reprogramming, with its inhibition leading to decreased H3K27me3 occupancy and activation of cardiac genes. However, this inhibition also reduces human iCMs incidence, conflicting with heart reprogramming goals (Tang et al., 2021). EZH2 is essential for epicardial cell migration by suppressing tissue inhibitor of metalloproteinase 3 (TIMP3), playing a critical role in cardiac development (Jiang et al., 2023).

While EZH2 in VSMCs is potential target for CVDs treatment, its mechanism and clinical applications require further investigation. Most studies have focused on animal models, with few using human cells. There is lack of experiments with EZH2-specific knock-out or knock-in in smooth muscle tissue to verify its role in disease. Consequently, significant challenges remain before clinical application.

As a single epigenetic regulator influencing multiple genes and pathways, EZH2 is an attractive target for complex diseases. However, many epigenetic modifying enzymes involved in diseases development remain unexplored, with unclear mechanisms. A better understanding of EZH2’s specific functions and regulatory mechanisms in cardiovascular system is necessary. Current research has primarily focused on EZH2’s role in VSMCs, with limited investigation in other cardiovascular cell types. Studies on the effects of EZH2-targeted therapy on different cell types and tissues will enhance our understanding of its role in CVDs and mitigate adverse effects from global EZH2 inhibition. For instance, EZH2 ablation in murine hearts causes various cardiovascular malformations and perinatal death, highlighting its crucial role in cardiovascular development (Chen et al., 2012). Additionally, EZH2 inhibition can affect hematopoietic and muscle stem cells, causing issues like blood disorder and impaired muscle repair (Juan et al., 2011; Mochizuki-Kashio et al., 2015). EZH2 inhibitor GSK-343 enhances human mononuclear cell reparative function post-myocardial infarction, thereby preventing infarct size expansion and cardiac dysfunction, by resolving H3K27 methylation and promoting inflammation resolution (Rondeaux et al., 2023). However, EZH2 inhibitor GSK126 increases vascular stiffness and elastin degradation, highlighting potential adverse cardiovascular effects (Ibarrola et al., 2024). Therefore, specific targeted inhibitors should be chosen for EZH2-targeted CVD therapy to minimize adverse effects.

Developing novel EZH2 inhibitors is crucial for improving therapy efficacy. Inhibition of EZH2 by UNC1999 significantly suppressed PDGF-BB-induced VSMC proliferation and neointima formation (Liu et al., 2020). In another study, PCSK9 inhibitor PEP2-8 trifluoracetate attenuates AS by regulating SNHG16/EZH2/TRAF5-mediated VSMC proliferation, migration and foam cell formation (Liu et al., 2023). Some EZH2 inhibitors, like DS-3201b, are in phase 2 clinical trials for solid tumors (Izutsu et al., 2023). Traditional Chinese medicines have demonstrated unique advantages in managing CVDs, and screening for EZH2 inhibitor among them is a feasible strategy (Li et al., 2020; Zhao et al., 2020; Wang et al., 2021; Li et al., 2022; Wu et al., 2022; Hou et al., 2024; Qi et al., 2024; Wang et al., 2024; Zhao et al., 2024). In personalized medicine, EZH2 can serve as a breakthrough point for individualized CVD treatment, enabling accurate diagnosis and treatment based on related biomarkers. Therefore, more preclinical studies and clinical trials are necessary to evaluate the potential application of EZH2 inhibitors CVDs. Current EZH2 inhibitors exhibit varying degrees of side effects and drug resistance (Duan et al., 2020; Adema and Colla, 2022). Consequently, further research on the three-dimensional structure and function of EZH2, as well as the screening for more selective and effective EZH2 inhibitors, is essential to provide better options for clinical application.

In conclusion, EZH2 plays a significant role in VSMCs proliferation, migration and survival. Despite its potential as a target for CVD treatment, many unresolved issues require further research. Future work should focus on elucidating EZH2’s mechanism, developing individualized treatment strategies, and creating novel inhibitors to advance CVD treatment and prevention. This will enhance our understanding and therapeutic capabilities, improving patient quality of life and reducing the global burden of CVDs.
